# Medical Education in London

**Published:** 1905-02-25

**Authors:** 


### Hospital Clinics.

### MEDICAL EDUCATION IN LONDON.

Sir Edward Fry's committee has dragged in by the heels the question which is agitating the University of London and medical circles at the present time. It affects the hospitals materially too because if the suggestions which the committee make are to be carried out, as we hope they may be carried out, there must be considerable saving in regard to the expenditure of the medical schools, under the present system. The patients too in the surgical wards of the hospitals will be saved from certain risks which were indicated by the President of the College of Surgeons in his evidence. For some time an effort has been made to raise £250,000 for the erection, equipment and endowment of a science school at South Kensington, in connection with London University, where medical students may pursue their studies for the first three years of their curriculum, prior to their entrance at the hospitals. Of this quarter of a million only £15,000 seems to have been promised, of which about half has been received. It would thus appear that public opinion is not in favour of a great expenditure of this kind for so limited a purpose as the provision of facilities for the earlier studies of medical students in the metropolis, during the first three years of their course. It is not surprising that this should be the case, and the originators of the South Kensington scheme seem to recognise the fact by the action they took at a meeting of the Senate of the University of London last week.

The Senate appointed a committee to confer with representatives of University College and King's College, and of the schools of the University and the Faculty of Medicine, as to the practicability of securing common courses of instruction in the preliminary and intermediate subjects of medical study at University College and King's College, such common courses to form part of a scheme for the concentration of the study of these subjects in the University.

This action synchronises with Sir Edward Fry's committee's support of the proposal that the senate should use its best endeavours to secure common courses of instruction for internal medical students in the preliminary and intermediate portion of their studies under appointed or recognised teachers at one or more centres. We understand that the two centres mentioned, University College and King's College, are anxious to secure the co-operation of the senate, so that they may be entrusted with the direction of the studies of the internal medical students referred to. Naturally, as each of these colleges has a hospital attached to it, objections must be raised by some of the authorities of the existing medical schools on the grounds that, if all the medical students of London are to spend their first three years at King's or University College, such an arrangement will naturally give an unfair advantage to King's and University College Hospitals in regard to the entries for clinical study. Such an objection is reasonable and natural, and if no alternative could be found it might necessitate the continuance of the effort in support of the South Kensington scheme.

It is of the greatest importance to the whole medical profession throughout the Empire and to the public generally that the present unsatisfactory and inadequate arrangements for medical instruction in the metropolis should be reorganised and improved. It is not creditable to the nation that the metropolis of the Empire, possessing as it does a greater opportunity for the study of disease, than any other city, should exhibit symptoms of decadence in this matter for want of organisation and equipment. London needs a system of concentration into one or two institutions of the smaller medical schools at present teaching physics, chemistry, biology, anatomy, physiology, pharmacology, materia medica, pathology, hygiene and public health, and forensic medicine. As the Commissioners pointed out in 1894 the number of students attending these several classes is very small, and there is often great difficulty in obtaining teachers properly qualified for the work. The establishment of provincial colleges and universities on a modern basis, with efficient teachers, has given a great impetus in their direction, with the result that some of the best teachers are being attracted to them, while the entry of medical students in London has steadily declined year by year for years and last year touched bottom. Hence the inclination of the smaller schools to concentrate has been quickened, though some of the larger ones may probably not come in to such a scheme at first. This last, as Sir Arthur Rücker pointed out, would tend to help rather than to retard a scheme of concentration, for it would be difficult to provide at first for the whole of the medical students of London in one institution. Hence the favour which has been shown at the moment to a scheme which aims at concentration in one or more centres in London.

We are glad to learn that the difficulty in regard to University College and King's College may be overcome by the action of the Royal College of Surgeons, which is contemplating the establishment of a centre of preliminary and intermediate study on a neutral basis for medical students in London.

On the motion of Mr. Makins, the Council has appointed a committee to consider and report upon the practicability of the institution by the College of a school for the teaching of the early and intermediate subjects of the medical curriculum and of advanced pathology. Such a scheme should command the support of the public in preference to the South Kensington proposals, because it must tend to economy in the original capital outlay, and also in the annual working expenses. No hospital would object, we presume, to support concentration at a centre worked and controlled by the Council of the College of Surgeons. Then too, as concentration means a considerable reduction in the present cost of the medical schools attached to the hospitals, considerable funds should be at once available for establishing an independent centre under the College of Surgeons This being so, having regard to the manifest failure of the South Kensington scheme in the pecuniary sense, and to the difficulties presented by the proposals in regard to University College and King's College as they stand, we hope that all parties may be willing to combine in favour of a great neutral centre established by the Royal College of Surgeons and Physicians, which can be organised and managed on an independent basis, so that each medical school may be guaranteed fair treatment in regard to the entry of medical students for the last two years of clinical study. We believe that such a settlement of this question would be found to work satisfactorily. It should secure that ultimately London will possess a medical school and a system of medical education worthy in all respects of the Empire and calculated to so raise the quality of medical education and medical knowledge that London may properly come to be recognised as the greatest school of medicine in the civilised world.

